# Weight perceptions, weight management practices, and nutritional status of emerging adults living in the Accra Metropolis

**DOI:** 10.1186/s40795-018-0265-4

**Published:** 2018-12-27

**Authors:** Esi Yaabah Quaidoo, Agartha Ohemeng, Margaret Amankwah-Poku

**Affiliations:** 1 Department of Nutrition and Food Science, College of Basic and Applied Sciences, University of Ghana, Accra, Ghana; 2 Department of Psychology, College of Humanities, University of Ghana, Accra, Ghana

**Keywords:** Weight perceptions, Emerging adults, Nutritional behaviours, Managing weight, Nutritional status

## Abstract

**Background:**

Many young people have a tendency to be concerned about their physical appearance and undertake practices in order to achieve certain body ideals. There is however limited information from developing countries on the weight perceptions of emerging adults (i.e. individuals leaving the adolescence life stage and preparing to take on adulthood) and whether these opinions influence their nutritional status and weight management practices. This study sought to assess emerging adults’ nutritional status, their weight perceptions and the methods they use to manage their weight.

**Methods:**

This study was cross-sectional, involving emerging adults (*N* = 192) recruited at shopping areas in the Accra Metropolis of Ghana. A pretested questionnaire was used to collect information on demographic characteristics, weight perceptions, and weight management strategies. Anthropometric measurements were taken using standard procedures. Descriptive analysis was performed on the demographic data, methods used to manage weight, and weight perceptions. Logistic regression was used to assess possible relationships between weight perceptions and nutritional status as well as weight perceptions and weight management practices.

**Results:**

The mean age of participants was 21.8(2.2) years with 51.0% of participants being female. Majority of the participants perceived normal weight status as the ideal body for themselves and half of them thought that they were slimmer than they actually were in reality. Three major weight management strategies were identified: engaging in physical activity, dieting and making lifestyle modifications (i.e. changes in normal eating habits coupled with regular physical activity and behavioral changes). Emerging adults who had an inaccurate body image perception were less likely (OR = 0.30, 95% CI: 0.15–0.61) to have a healthy nutritional status than emerging adults who had an accurate body image perception.

**Conclusion:**

Weight perception was associated with nutritional status. Discussions with nutrition professionals regarding realistic weight ideals would be beneficial for this age-group since half of the study’s participants had inaccurate perceptions about their current weight statuses even though their statuses were normal.

**Electronic supplementary material:**

The online version of this article (10.1186/s40795-018-0265-4) contains supplementary material, which is available to authorized users.

## Background

Individuals between the ages of 18 to 25 can be referred to as emerging adults [8, 29, 32]. Emerging adulthood is a life-stage set apart from any other life-stage since one cannot be referred to as an adolescent since adolescence is characterized by dependency on caretakers and neither can one be referred to as a full-fledged adult since adulthood is characterized by enduring social responsibilities and self-sufficiency which typically has not been attained at emerging adulthood [8]. Individuals going through emerging adulthood are usually faced with various life circumstances where they need to make significant adjustments while learning to cope with new life situations. Many emerging adults, for the first time, are responsible for their meal preparations and other nutritional behaviours. The period is also characterized by individuals forming their own opinions on different life ideologies including what is considered physically attractive. Physical attractiveness is a valuable tool in many situations of human interaction, as such, many emerging adults tend to be concerned about their physical appearance and are keen to change or maintain it [18]. There has been high interest in body ideals and how weight is perceived around the world, with several studies documenting a prevalence of different body weights as idyllic in different parts of the world [6, 12, 13, 20, 28, 42]. Some studies report weight perceptions in adolescents [3–5, 10, 25, 39] but not as much report on emerging adults [14, 26, 28, 29]. Majority of these studies however, have come from outside of Africa.

While the weight of an individual contributes significantly to one’s physical appearance [35], sociocultural factors such as collectivist ideologies have the ability to influence weight preference cultivation [37]. Abubakari et al., [1], observed that West Africans traditionally appreciated body types that equated what is referred to as the overweight body type in developed countries yet Benkeser, Biritwum, & Hill, [9] who studied older West African women, indicated that almost half of participants preferred a slimmer body size. Young people tend to want to live up to the standards of beauty set by the society they belong to in order to avoid stigmatization which comes along with being a certain weight status (overweight or underweight). This desire may lead to a search for the perfect body using various weight management techniques [6, 17]. When one seeks to maintain or alter one’s weight, individuals are encouraged to seek the healthiest options to achieve set weight goals and keep a healthy nutritional status in the process. With the race-specific link between body size and beauty [6] there is the need to understand nutritional behaviours such as weight perceptions of individuals within specific age demographics in order to create public health interventions that are innovative, population specific and culturally acceptable for healthy weight promotion within communities. Additionally, when a person has a realistic and positive attitude about their body, they tend to show appreciation, respect and acceptance for themselves and as such these individuals are more likely to go about managing their weight in healthy manners [7, 28, 35].

Given the unique life circumstances emerging adults face, this study sought to assess the nutritional status, weight perceptions and the weight management practices of a sample of emerging adults in an urban African capital.

## Methods

The study population used in Quaidoo, Ohemeng & Amankwah-Poku’s [34] report on the sources of nutrition information and level of nutrition knowledge among young adults in the Accra metropolis is the same study population used in this report. The study took place in the Accra metropolis in the Greater Accra Region of Ghana. Two shopping centres (Accra Mall and Makola Market) were randomly selected from a list of shopping areas (malls and markets) that are located in the metropolis at the time of this study. The target population was individuals (both sexes) between the ages of 18 to 25 because of the observations that this age group experiences significantly more freedom to choose when it comes to their nutrition choices than prior years [31]. Ethical approval for this study was obtained from the Ethics Committee for Basic and Applied Science (ECBAS), University of Ghana (ECBAS 006/16–17). Permission to collect data from the Accra mall was obtained from management of the mall. Makola market is an open market, thus no formal permission was required.

Participants were recruited using convenience sampling method. Convenience sampling was used in order to meet sample size requirement within the stipulated data collection period. Hence, the responses of study participants do not necessarily represent the entire emerging adult population. Stands were set up at both study sites and the objectives of the study were thoroughly explained to prospective participants who were approached by the researchers. Individuals were included if they were between the ages of 18 to 25 years, had completed at least their junior high school education, and were residing in the Accra metropolis at the commencement of the study. Individuals with symptomatic diseases, pedal oedema, who were physically disabled or pregnant, were excluded from the study as this would have affected the accuracy of anthropometric measurements. Prospective participants were enrolled into the study only after they agreed to participate and had signed an informed consent form.

Weight and height measurements were taken as per standard procedures [16]. The height of study participants was taken using a portable wall-mounted Seca GmbH & Co.KG. 2,171,821,009 Stadiometer; participants stood upright with their back against a wall and their head in the Frankfurt horizontal plane while the researcher and one field assistant took the height measurement. The weight was measured using an Ohaus SD 200 digital weighing scale; participants wore light clothing and were barefoot or wearing light socks whilst their weight and height were being measured. The waist and hips circumferences were measured using a constant tension tape as per standard procedures [41]. Participants were asked to stand with their feet together and place their arms at their side with the palms of their hands facing inwards for both waist and hips circumference measurements. For waist circumference measurements, the inferior margin of the last rib and the crest of the ilium were located then the midpoint was measured with the tension tape measure. The tension tape was placed over the midpoint and wrapped around each participant. The participant was asked to breathe out gently before the measurement was read at the level of the tape to the nearest 0.1 cm and recorded. For the measurement of the circumference of the hips, the tape was placed around the maximum circumference of the buttocks and checked that the tape was horizontal all around the body. The measurement was then read at the level of the tape to the nearest 0.1 cm and recorded. A pretested questionnaire was used to obtain demographic information, weight perceptions, as well as weight management practices. The questionnaire was interviewer-administered and on average 20 min was spent on each participant Additional file 1.

In assessing weight perceptions, the Pulvers’ figure rating scale [33] was shown to participants. This standardized figure rating scale was used because of its cultural relevance as a body image rating instrument for people of African descent [33]. Participants were presented with a series of nine male and nine female silhouette pictures that depicted body sizes starting from very thin (assigned ‘1’) and ending at morbidly obese (assigned ‘9’). The pictures were arranged in two rows. The top row depicted male body sizes while the one below represented body sizes for females. Participants were required to circle one of the nine silhouettes that fit the participant’s idea of what their current body looks like, the body type the participant wants for themselves, and the body type the participant thought Ghanaian society wants for their sex. In the section that assessed weight management practices, participants were asked whether they were currently trying to do anything about their weight. Further questions were posed to identify any approaches or treatments actively being used by a participant to alter or manage weight in order to properly classify their weight management strategy.

Statistical package for social scientists (SPSS) 20.0 software was used to analyze all data at 95% confidence interval. Demographic data, weight perceptions and weight management strategies underwent descriptive analysis.

The assignment of body weight status on the Pulver’s figure rating scale was as follows: underweight (silhouettes 1 and 2, scored as 1), normal weight (silhouettes 3, 4 and 5, scored as 2), overweight (silhouettes 6 and 7, scored as 3) and obese (silhouettes 8 and 9, scored as 4). To assess weight perception, the Feel-weight-status-minus-Actual-weight-status Index (FAI) was used to represent participants’ weight perception [42]. FAI is an index used to assess how realistic a weight status perception of a participant is on the basis of body size assessment (i.e. Actual-weight-status) and the feel figure (i.e. the silhouette a participant picked as their opinion of what their current body looks like). FAI was assessed as follows: the BMI of a participant was referred to as their ‘Actual-weight-status’. Actual-weight-status was categorized based on WHO’s cut-points for adults: underweight (BMI below 18.5 kg/m^2^, scored as 1), normal weight (BMI from 18.5 kg/m^2^ to 24.9 kg/m^2^, scored as 2), overweight (BMI from 25 kg/m^2^ to 29.9 kg/m^2^, scored as 3), and obese (above 29.9 kg/m^2^, scored as 4) [40]. Scores were calculated by subtracting the actual-weight-status score from perceived current body weight score (i.e. feel-weight-status). This conventional code was subtracted from the perceived current body weight score. An overall score of zero indicated accurate body image perception. Positive scores indicated that participants perceived that they were heavier (fatter) than they actually were, whereas negative scores indicated that individuals perceived that they were thinner than they actually were in reality.

Binary logistic regression analysis was used to examine the possible relationship between weight perceptions and the nutritional status of participants in this study. FAI, the main independent variable, was coded as inaccurate body image perception and accurate body image perception. Nutritional status was presented as two separate dependent variables: BMI (coded as unhealthy BMI {underweight/overweight/obese} and healthy BMI) for one test; and waist-to-hip ratio (unhealthy waist-to-hip ratio {women i.e. above 0.8, men i.e. above 0.9} and healthy waist-to-hip ratio) for another test. Logistic regression was also conducted to examine the possible association between weight perception (FAI) and weight management strategies. Weight perceptions was the independent variable and weight management practices was the dependent variable of interest (coded as ‘holistic’ weight management practices {i.e. making lifestyle modifications} and ‘other’ weight management practices {i.e. physical activity or dieting}).

## Results

A hundred and ninety-two emerging adults took part in this study. Table 1 shows the demographic characteristics of these emerging adults including their occupations which were classified based on the International Labour Organization’s standards of classifying occupations [21]. Most of the study’s participants were students (66.1%) and had completed a senior high school level education. About half (51%) of the study’s participants were female and the mean age was 21.8 **±** 2.2 years.

**Table 1:** Demographic classification of participants (*n* = 192)

Variable	Mean (SD)	*n* (%)
Age (years)	21.8 (2.2)	
Ethnicity		
Akan		94 (49.0)
Ewe		45 (23.4)
Ga-Adangbe		40 (20.8)
Northerner		13 (6.8)
Occupation^a^		
Student		127 (66.1)
Services/Sales workers		23 (12.0)
Professionals		19 (9.9)
Crafts and related trades workers		9 (4.7)
Clerical support workers		3 (1.6)
Elementary Occupation		1 (0.5)
Unemployed		5 (2.6)
Other^b^		5 (2.6)
Highest Qualification		
Senior high school		127 (66.1)
Post-secondary school^c^		44 (22.9)
Junior high school		21 (10.9)

^a^Aside from ‘student’, occupation of participants was categorized based on the International Standard Classification of Occupations (ISCO) by the International Labour Organization (ILO) [21]

^b^Other occupations reported were footballer and actor/actress

^c^Includes clerical, vocational, polytechnic and university institutes

**Fig. 1:**
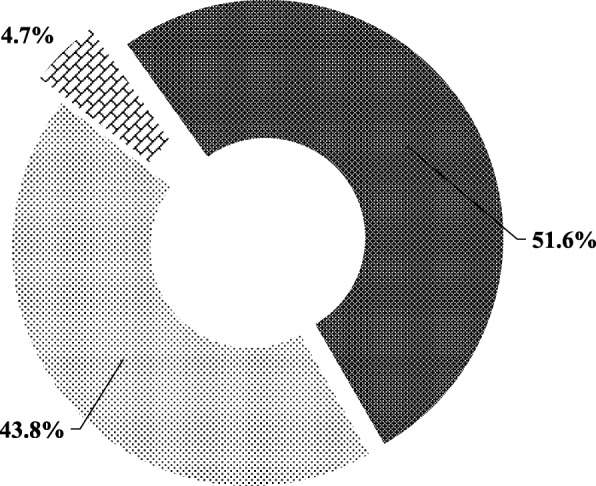
Weight Perception of study participants (*N* = 192). Thin polka dot pattern indicates participants who perceived their weight as their actual weight status. Brick pattern indicates participants who perceived their weight to be greater than their actual weight status (they thought that they were fatter than they actually are in reality). Dense polka dot pattern indicates participants who perceived their weight to be less than their actual weight status (they thought that they were thinner when in fact they were heavier)

**Fig. 2:**
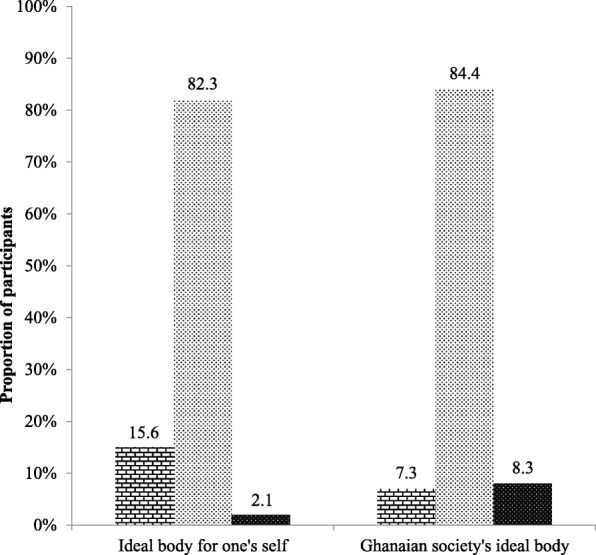
Participants’ perceptions on body ideals; (*N* = 192). Brick pattern indicates the proportion of study participants who selected an underweight status silhouette regarding their perceptions on body ideals. The thin polka dot pattern indicates the proportion of study participants who selected a normal weight status silhouette whilst the thick polka dot pattern indicates the proportion of participants who selected an overweight status silhouette as their ideal

About two-thirds of participants i.e. 69.3% (133 participants out of the total sample size of 192) had normal BMI. Less than a quarter of participants i.e. 23.4% (45 participants out of 192) were overweight using BMI, but about 34% (i.e. 65 participants out of 192) were classified as overweight based on Waist-to-Hip Ratio (WHR). Figure 1 shows participants’ body image discrepancies using Feel-weight-status-minus-Actual-weight-status Index (FAI) and 51.6% of the participants (i.e. 99 participants out of 192) perceived that they were thinner than they actually were in reality. Interestingly, less than half of the participants i.e. 43.8% (84 participants out of 192) had an accurate self-assessment of their weight even though more than half i.e. 69.3% (133 participants out of 192) had a normal BMI. Majority of participants i.e. 82.3% (158 participants out of 192) selected normal weight silhouettes as their idea of the perfect body for themselves (Fig. 2), and a similar proportion i.e. 84.4% (162 participants out of 192) selected normal weight silhouettes as what they perceived Ghanaian society idealized as the perfect body for their sex.

Significant factors that were associated with nutritional status in the study sample were sex, age, and weight perception (FAI) of participants (Table 2). Participants who had an inaccurate body image perception were 70% less likely to have a healthy nutritional status compared to those who had an accurate body image perception (95% CI: 0.15–0.61). Also, male participants were almost 5 times more likely to have a healthy nutritional status (WHR) as compared to female participants (95% CI: 2.19–10.12). Participants aged 18 to 21 were 40% less likely to have a healthy nutritional status (WHR) when compared to participants aged 22 to 25 (95% CI: 0.18–0.93).

**Table 2:** Relationship between weight perception and nutritional status of emerging adults (*n* = 192)

Variable	^a^Normal weight status		^b^Normal WHR	
	Odds ratio	95%CI	Odds ratio	95%CI
Sex				
Male	1.3	0.74–2.59	4.7**	2.19–10.12
Female	1		1	
Age				
18–21 year olds	0.6	0.30–1.41	0.4**	0.18-0.93
22–25 year olds	1		1	
FAI^c^				
Inaccurate perception	0.3**	0.17–0.67	0.6*	0.29–1.11
Accurate perception	1		1	

^a^Refers to Body Mass Indices which indicate normal weight status i.e. 18.5 kg/m^2^ to 24.9 kg/m^2^

^b^Refers to Waist-to-hip ratios which indicate normal central adiposity i.e. ratio below 0.8 for females and 0.9 for males

^c^Refers to the Feel-weight-status-minus-Actual-weight-status Index i.e. inaccurate perception equates perceiving one’s weight as greater than it actually is or perceiving one’s weight as less than it actually is. Accurate perception equates perceiving one’s weight as what it actually is

*p-value < 0.1, **p-value < 0.05

**Fig. 3:**
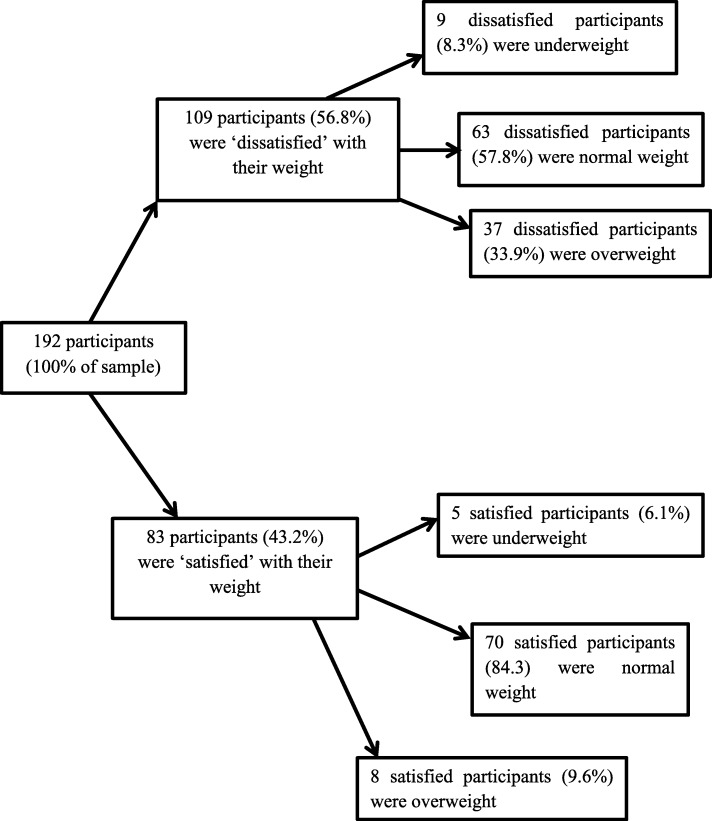
Participants’ personal concerns on their current body weight; (*N* = 192). Breakdown of study participants’ opinions of their current weight statuses and the BMI category they belonged to at the time of data collection

A total of 118 participants out of the study’s sample of 192 participants reported actively managing their weights at the time of the interview i.e. 61.5% of the total sample even though 109 participants i.e. 56.8% expressed dissatisfaction with their current body weight (Fig. 3). Three main strategies were identified by participants and these were: 39.0% were engaging in physical activities (i.e. 46 participants out of 118), 25.4% were undertaking lifestyle modifications (i.e. 30 participants out of 118) and 35.6% were actively dieting (42 participants out of 118). Table 3 shows that there was no significant relationship between weight perception and weight management strategies. The only significant predictor of using a holistic approach to weight management was employment status. Employed participants were about five times more likely to use a holistic weight management strategy (i.e. lifestyle modifications) than unemployed emerging adults (95% CI: 1.22–17.47).

**Table 3:** Relationship between weight perception and weight management strategies (*n* = 118)

Variable	^a^Holistic weight management strategy	
	Odds ratio	95%CI
Sex		
Male	2.0	0.70–5.88
Female	1	
Employment Status		
Employed	4.6**	1.22–17.47
Unemployed	1	
Feeling about weight		
Dissatisfied	0.4	0.12–1.47
Satisfied	1	
FAI^b^		
Inaccurate perception	0.8	0.36–1.91
Accurate perception	1	

^a^Refers to the use of lifestyle modifications (a combination of diet, physical activity and behavioural changes) as the weight management strategy used i.e. holistic weight management strategy equates making lifestyle modifications whilst other weight management strategy equates using dieting only or physical activity only

^b^Refers to the Feel-weight-status-minus-Actual-weight-status Index i.e. inaccurate perception equates perceiving one’s weight as greater than it actually is or perceiving one’s weight as less than it actually is. Accurate perception equates perceiving one’s weight as what it actually is

**p-value < 0.05

## Discussion

The body image discrepancy assessment of participants revealed that half of the participants thought that they were thinner than they actually were in reality. This finding is consistent with the observation made by Benkeser et al., [9], who reported that almost half of their study participants preferred a slimmer body size. Renzaho et al., [37], reported that Blacks were less likely than other racial groups to perceive themselves as ‘fatter’ than they actually are in reality and this may be due to the fact that many Blacks belong to collectivist cultures where positive feedback about being ‘heavy’ is given by friends and family even though they are clearly not ‘thin’. This may encourage a person to believe that they are slender-looking when in fact they are not. It was interesting to find that there was an appreciation for normal weight silhouettes because typical West African culture, as stated by Abubakari et al., [1], favours overweight figures. Both Poobalan & Aucott, [32], and Renzaho et al., [37], pointed out that collectivist societies (many of which are sub-Saharan African nations) show preferences for a full-bodied physique, and a rounded body is the norm for both men and women. In this study, however, most study participants perceived normal weight statuses as Ghanaian society’s current ideal. Perhaps there is a change in the traditional Ghanaian preference to a leaner frame due to there being more opportunities for social comparison and constant surveillance of body parts through online resources. Many young Ghanaians have a heavy online presence [30, 34] and this may have exposed them to different cultures and perspectives; pictures of people- celebrities to normal individuals on frequented online platforms can have an impact on a young person’s perception of what it means to be ‘beautiful’ [18, 43]. Coetzee et al., [17], stated that with recent political and economic progress in Africa, this may just have altered body size preferences among young Africans who are being exposed to more westernized ideas. Adusei (Adusei: The relationship between Obesity and depressive symptoms among young Ghanaians, unpublished), [2], made mention in her work that overweight individuals were at times taunted as ‘oboshie’ or ‘obolo’- words which translate into ‘fat person’ in the local Ghanaian dialect- and that these names may be stigmatization tools which may deter a modern-day young person from thinking that being overweight is beautiful.

More than half of the study’s participants indicated that they were dissatisfied with their current weight status hence they were actively trying to alter their weight status but some participants who stated they were satisfied with their current weight status were also actively trying to alter their weight status. Three major weight management strategies were identified: physical activity, dieting, and lifestyle modifications. The proportions of study participants who reported using physical activity and dieting to manage their weight were similar. Even though making lifestyle modifications (changes in diet coupled with physical activity and a strong psychosocial support system to enable behavioural changes) is recommended since it is a holistic approach to weight management [11, 15], it was the least utilized weight management strategy. Making lifestyle modifications requires consistency and commitment. Focusing on only changing physical activity regimens or only dietary habits may not render sustainable weight alteration results because once the physical activity or the diet is stopped, there is the possibility that the original weight will recur [11].

Although Sirang et al., [38], reports that how a young person perceives weight reflects on their weight management behaviour, in the present study, there was no significant relationship between weight perception and the type of weight management behaviour employed by participants. However, participants who were employed were five times more likely to carry out a holistic weight management strategy than unemployed study participants. With a reliable source of funds that is enjoyed by the employed, perhaps it would be easier for an emerging adult to undertake the details of making lifestyle modifications as compared to an emerging adult whom is unemployed and may be experiencing financial constraints. According to Laborde, [22], steady wages influence weight management behaviours such as purchasing gymnasium membership and using it regularly. Also, consistent healthy eating habits and minutes spent exercising increased among Laborde’s [22] study participants as their income increased. It must however be noted that regular adequate physical activity can be achieved without financial cost.

Participants who reported using physical activities as a weight management strategy indicated that they played sports, skipped, jogged or performed exercises that were aimed at toning the stomach muscles. Some female participants reported doing stomach exercises with a waist-trainer on to achieve their weight goals, a method which may have negative consequences. A waist-trainer is a waist cinching garment that is wrapped around the mid-section of the body to encourage a thinner waistline appearance to form with time due to constriction. Healthcare professionals advise against the excessive use of waist-trainers because extended use can weaken the abdominal muscles and cause the deformation of certain organs such as the stomach, liver and lungs [24]. Thus, this method of exercising may not be a helpful one in the long term. Physical activity alone may be helpful to manage one’s weight since consistently active individuals are more likely to achieve weight maintenance and have a healthy body composition. However, studies have demonstrated that a combination of regular physical activity and a healthy well-balanced diet (regardless of whether one is trying to gain, lose or maintain their weight) produce sustainable results compared to practicing one method alone [11, 19, 23, 36].

Some participants used food restriction (i.e. starvation or skipping meals) as a means to attain their weight goals. According to Malinauskas et al., [27], one of the unhealthiest weight management strategies include not eating any food for significant periods of time and skipping meals intentionally as this slows down the body’s normal metabolic activity. In this instant, temporal weight loss occurs, but with the reversion to old eating habits weight regains are inevitable and at times larger than before (this is termed weight cycling). Weight cycling is even more harmful than being persistently overweight because studies have shown that it is positively associated with higher chances of developing cardiovascular diseases [11].

## Conclusion

This study found that weight perception was associated with nutritional status among emerging adults in the Accra Metropolis, Ghana. Open discussions with nutrition professionals regarding healthy weight and effective weight management practices would be beneficial for this age-group since many participants had inaccurate perceptions regarding their actual weight status and were actively trying to alter their weight even though they had normal weight statuses.

## Additional file

Additional file 1: Research Questionnaire. (DOCX 394 kb)

## Abbreviations

BMI: Body Mass Index
CDC: Centers for Disease Control and Prevention
ECBAS: Ethics Committee for Basic and Applied Science
FAI: Feel-weight-status-minus-Actual-weight-status Index
SPSS: Statistical package for social scientists
WHO: World Health Organization
WHR: Waist-to-hip ratio
