# Supplementary material for: Weight perceptions, weight management practices, and nutritional status of emerging adults living in the Accra Metropolis
Source: BMC Nutr. 2018 Dec 27;4:53. doi: 10.1186/s40795-018-0265-4 (PMC7050933; doi:10.1186/s40795-018-0265-4)
Supplement: Supplementary file 1 — Research Questionnaire. (DOCX 394 kb) [file 40795_2018_265_MOESM1_ESM.docx]

**Research Questionnaire**

Thank you for taking the time to fill this questionnaire. We will keep your responses confidential so please answer these questions as honestly as possible. There is no right or wrong answer.

1. What is your gender?
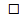
Male
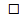
Female
2. What is your ethnicity …………………………………..
3. What is your occupation? …………………………………………
4. How old are you? …………………… Date of birth: ………………………………
5. What is your marital status ……………………………….
6. What is your highest education qualification ……………………………………………

**Weight Management Practices and Lifestyle:**

**Please write the code, attached to your option, inside the brackets and fill in the spaces where applicable**:

1. Do you have any concerns about your current body weight?

0= No 1= Yes **[ ]**

1. How do you feel about your size?

…………………………………………………………………………………………………

1. Who talks the most about your weight? ……………………………………………………....
2. I am currently trying to…..

0= Not trying to do anything about my weight 1= Lose weight 2= Gain weight

3= Maintain my current weight **[ ]**

1. Within the past 30 days, I have ………**[ ]**……. in order to achieve my weight goals

0= Dieted 1= undergone surgery 2= engaged in physical activities (exercising) 3= made lifestyle modifications 4= used medicine 5= done nothing

1. In regard to question 11, I got information to use this method from…………………………...
2. This method is working for me

0= No 1= Yes 2= I do not know 3= not applicable **[ ]**

1. This source of information (indicated in question 12) has affected the way I feel about my body

0= No 1= Yes 2= I do not know 3= not applicable **[ ]**

1. During the past 30 days, I intentionally ate less food, fewer calories, or foods low in fat to lose weight or to keep from gaining weight

0= No 1= Yes **[ ]**

1. a) Do you drink alcohol? 0= No 1= Yes **[ ]**

b) If ‘yes’, in a typical week, how many times do you drink alcoholic beverages

0= 1-2 times 1= 3-4 times 2= 5-7times 3=more than 7 times **[ ]**

1. a) Do you smoke? 0= No 1= Yes **[ ]**

b) If ‘yes’, in a day I smoke

0= less than half a pack 1= half a pack 2= about a pack 3= more than one pack **[ ]**

1. How many days a week do you exercise **at least** 20 minutes, to the point that you sweat or breathe hard?

0= Never 1=1-2 times a week 2=3-4 times a week 3=5-7 times a week 4=7 times per week **[ ]**

1. How many days a week do you exercise **at least** 30 minutes, to the point that you sweat or breathe hard?

0= Never 1=1-2 times a week 2=3-4 times a week 3=5-6 times a week 4=7 times per week **[ ]**

1. How many days a week do you exercise **at least** 60 minutes, to the point that you sweat or breathe hard?

0= Never 1=1-2 times a week 2=3-4 times a week 3=5-6 times a week 4=7 times per week **[ ]**

1. During the past 30 days, did you intentionally go without eating for 24 hours or more (also called fasting) with the motive to lose weight or to maintain weight? 0= No 1= Yes **[ ]**

1. During the past 30 days, did you take any diet pills, powders or liquids without a doctor's advice to lose weight, gain weight or to keep from gaining weight? 0= No 1= Yes **[ ]**

**Weight Perceptions**

Under this section, please circle the number beneath the silhouette that best represents your opinion and remember that there is no right or wrong answer.

1. Which picture do you think best represents your body currently? **Please circle.**


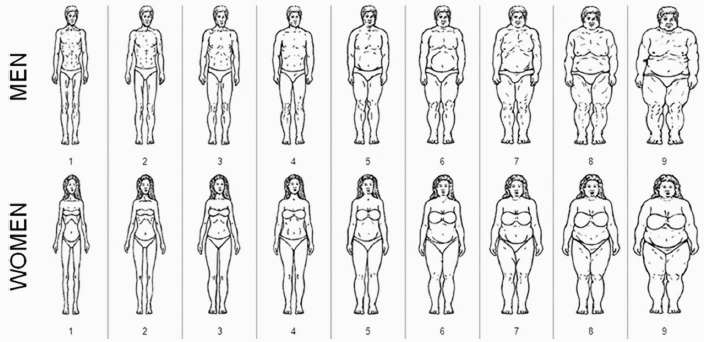


41. Which silhouette do you think best represents the body you want to have? **Please circle**.
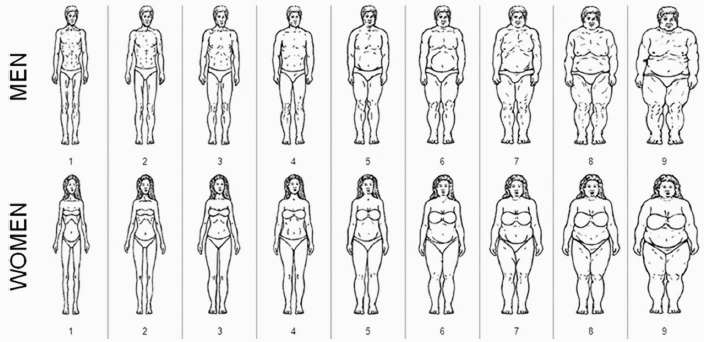


42. Which picture do you think represents the ideal body for your gender in Ghanaian society? **Please circle.**


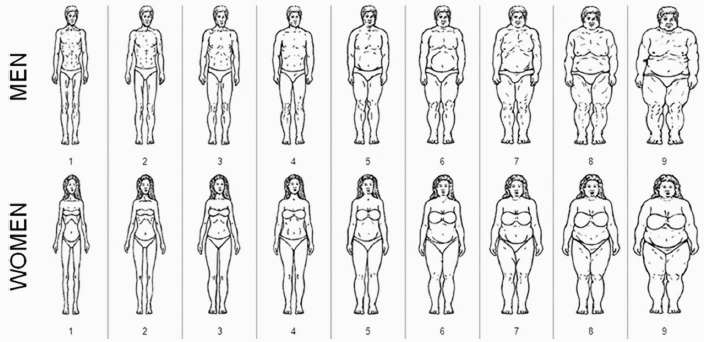


*For Researcher & Field assistants

Measured weight= Measured height= Calculated Body Mass Index= Measured waist circumference= Measured hip circumference= WHR=

## ****Score Sheet for research questionnaire****

(Not to be shown to participants; for researcher’s use only)

Weight Perceptions:

Pulvers’ figure rating scale categories for silhouettes:
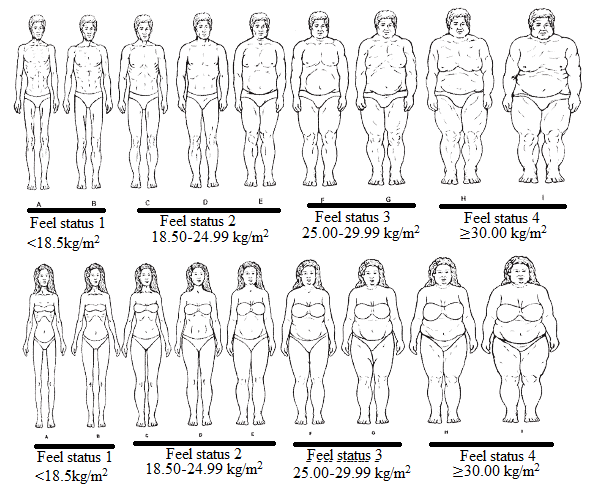


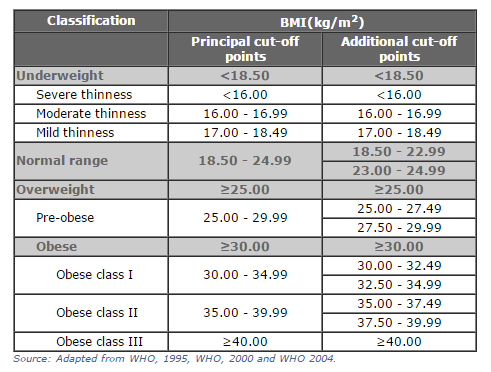


Feel-Weight-Status minus Actual-Weight-Status Index (FAI) for body satisfaction assessment:

Actual-Weight-Status based on measured BMI of participant:

- underweight [BMI below 18.5kg/m^2^] = Conventional code 1
- normal weight [BMI=18.5 kg/m^2^ – 24.9 kg/m^2^] = Conventional code 2
- overweight [BMI= 25 kg/m^2^ -29.9 kg/m^2^ ] = Conventional code 3
- obese [BMI= 30 kg/m^2^ and above] = Conventional code 4

Perceived current body weight i.e. Feel-Weight-Status:

- Silhouettes a and b for feel status 1 i.e. very thin/thin= Conventional code 1
- Silhouettes c, d and e for feel status 2 i.e. average = Conventional code 2
- Silhouettes f and g for feel status 3i.e. slightly heavy/overweight= Conventional code 3
- Silhouettes h and i for feel status 4 i.e. obese = Conventional code 4

Therefore,

FAI= Feel-Weight-Status’ conventional code (subtracted from) Actual-Weight-Status conventional code

Scores range from -4 to +4

Accurate body image perception = 0

Perception that one is heavier than they actually are = +1 to +4

Perception that one is thinner than they actually are = -4 to -1
